# Supplementary material for: Comparative multiomics analysis of cell physiological state after culture in a basket bioreactor
Source: Sci Rep. 2022 Nov 23;12:20161. doi: 10.1038/s41598-022-24687-4 (PMC9686226; doi:10.1038/s41598-022-24687-4)
Supplement: Supplementary file 1 — Supplementary Information 1. [file 41598_2022_24687_MOESM1_ESM.zip › raw data/Transcriptomic raw data/6.Enrichment/1.GO/TE1vsTC1/UP/TE1vsTC1.up.GObp_DAG.pdf]

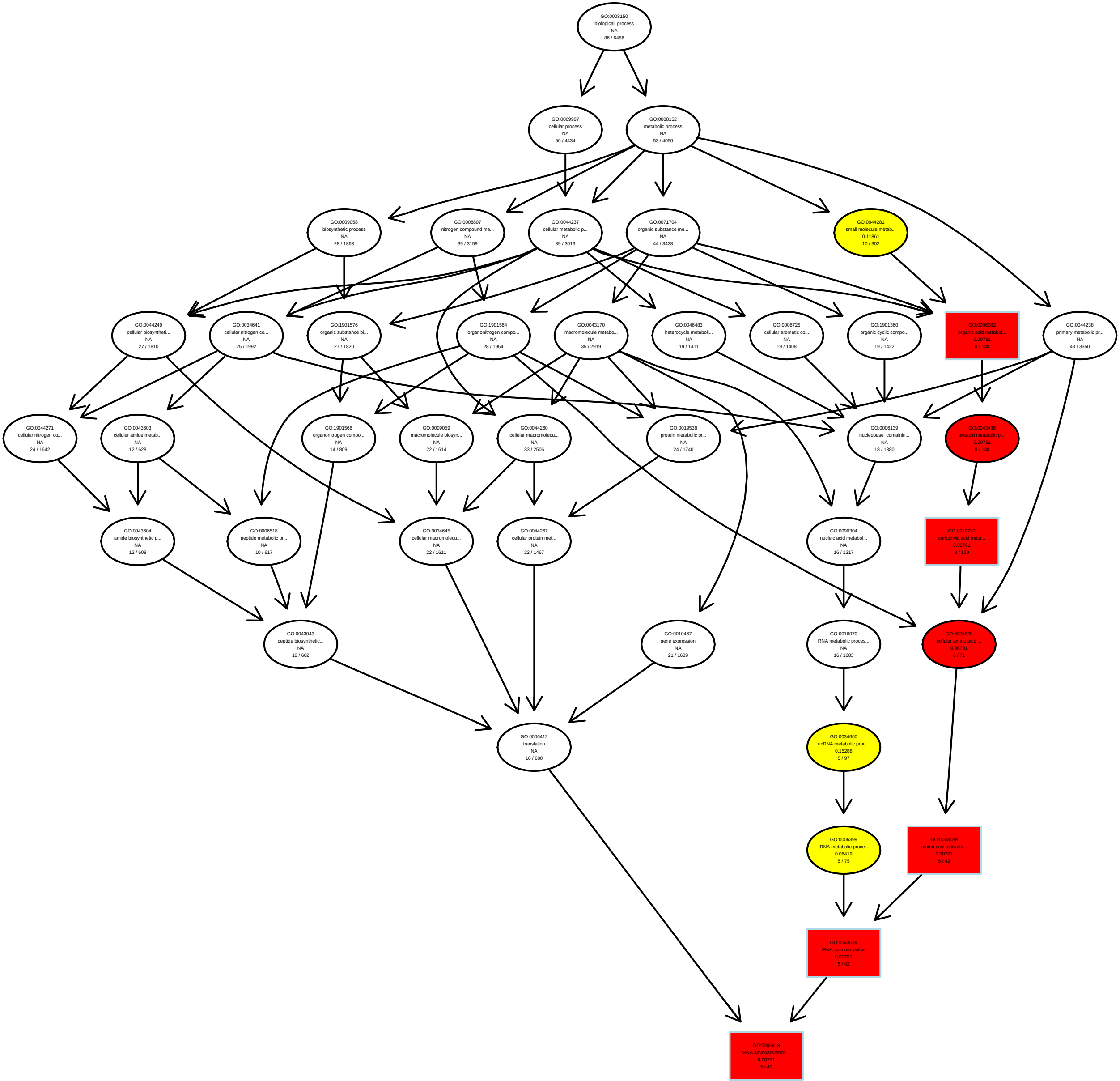
GO:0008150 biological_process NA 86 / 6486
GO:0009987 cellular process NA 56 / 4434
GO:0008152 metabolic process NA 53 / 4050
GO:0009058 biosynthetic process NA 28 / 1863
GO:0006807 nitrogen compound me... NA 39 / 3159
GO:0044237 cellular metabolic p... NA 39 / 3013
GO:0071704 organic substance me... NA 44 / 3428
GO:0044281 small molecule metab... 0.11861 10 / 302
GO:0044249 cellular biosyntheti... NA 27 / 1810
GO:0034641 cellular nitrogen co... NA 25 / 1992
GO:1901576 organic substance bi... NA 27 / 1820
GO:1901564 organonitrogen compo... NA 28 / 1954
GO:0043170 macromolecule metabo... NA 35 / 2919
GO:0046483 heterocycle metaboli... NA 19 / 1411
GO:0006725 cellular aromatic co... NA 19 / 1408
GO:1901360 organic cyclic compo... NA 19 / 1422
GO:0006082 organic acid metabol... 0.00791 8 / 130
GO:0044238 primary metabolic pr... NA 43 / 3350
GO:0044271 cellular nitrogen co... NA 24 / 1642
GO:0043603 cellular amide metab... NA 12 / 628
GO:1901566 organonitrogen compo... NA 14 / 809
GO:0009059 macromolecule biosyn... NA 22 / 1614
GO:0044260 cellular macromolecu... NA 33 / 2506
GO:0019538 protein metabolic pr... NA 24 / 1740
GO:0006139 nucleobase−containin... NA 18 / 1380
GO:0043436 oxoacid metabolic pr... 0.00791 8 / 130
GO:0043604 amide biosynthetic p... NA 12 / 609
GO:0006518 peptide metabolic pr... NA 10 / 617
GO:0034645 cellular macromolecu... NA 22 / 1611
GO:0044267 cellular protein met... NA 22 / 1467
GO:0090304 nucleic acid metabol... NA 16 / 1217
GO:0019752 carboxylic acid meta... 0.00791 8 / 129
GO:0043043 peptide biosynthetic... NA 10 / 602
GO:0010467 gene expression NA 21 / 1639
GO:0016070 RNA metabolic proces... NA 16 / 1083
GO:0006520 cellular amino acid ... 0.00791 6 / 71
GO:0006412 translation NA 10 / 600
GO:0034660 ncRNA metabolic proc... 0.15288 5 / 97
GO:0006399 tRNA metabolic proce... 0.06419 5 / 75
GO:0043038 amino acid activatio... 0.00791 5 / 42
GO:0043039 tRNA aminoacylation 0.00791 5 / 42
GO:0006418 tRNA aminoacylation ... 0.00791 5 / 40
